# Hemobezoar, a rare cause of intestinal obstruction after mini gastric bypass/one anastomosis gastric bypass: case report

**DOI:** 10.1093/jscr/rjaf610

**Published:** 2025-08-12

**Authors:** Jaime Ponce De Leon, Mauricio Fabian Palacios, Alex Paul Guachilema Ribadeneira, David Gambino, Felipe Ortiz

**Affiliations:** Department Bariatric and Metabolic Surgery, Instituto Nefrologico de Tijuana Misión de San Ignacio 4428, Zona Urbana Rio Tijuana, Tijuana B.C. 22010, México; Department Bariatric Surgery, Hospital Metropolitano Av. Mariana de Jesús, s/n, Quito, Pichincha, 170521, Ecuador; Servicio de Cirugía General y Laparoscopica, Hospital General Enrique Garces Av. Chilibulo y Enrique Garcés, Quito 170131, Ecuador; Departamento de Medicina Bariatrica, Instituto Nefrologico de Tijuana, Misión de San Ignacio 4428, Zona Urbana Rio Tijuana, Tijuana, B.C. 22010, México; Servicio Cirugia Bariatrica, Hospital Universitario del Rio, Avenida 24 de Mayo, y, Calle Cuenca, 010109, Cuenca, Ecuador

**Keywords:** hemobezoar, bariatric surgery, mini gastric bypass, one anastomosis gastric bypass, bariatric laparoscopy, intestinal obstruction

## Abstract

Hemobezoar is a rare cause of intestinal obstruction after mini gastric bypass/one anastomosis gastric bypass. Clot formation is often caused by bleeding in the anastomotic staple line. Symptoms include abdominal pain, vomiting, and abdominal distension. Surgical treatment should be performed promptly to prevent serious complications, such as anastomotic leaks, intestinal perforation, and death. Intraoperative endoscopy is useful for examining intraluminal mucosa and identifying the cause of the obstruction. This report describes a case of hemobezoar in a male patient. Few cases of hemobezoar have been reported in the literature.

## Introduction

Mini gastric bypass/one anastomosis gastric bypass (MGB/OAGB) can effectively promote weight loss and reduce comorbidities [1]. MGB-OAGB is also performed in cases of surgical failure and complications. The reported rate of complications of MGB-OAGB is 3.4% [2]. Similar to other bariatric procedures, MGB/OAGB can lead to complications, including afferent loop syndrome, anastomotic obstruction, and severe diarrhea. Surgical revision is necessary in patients with anastomotic hemorrhage, perforations, and stenosis [2].

Although the etiology of intestinal obstruction is multifactorial, this surgical emergency is rarely caused by intraluminal blood clots [3]. This report describes a case of a man who underwent MGB-OAGB and presented with intestinal obstruction from a blood clot at the anastomosis site in the immediate postoperative period.

## Case presentation

**Figure 1:**
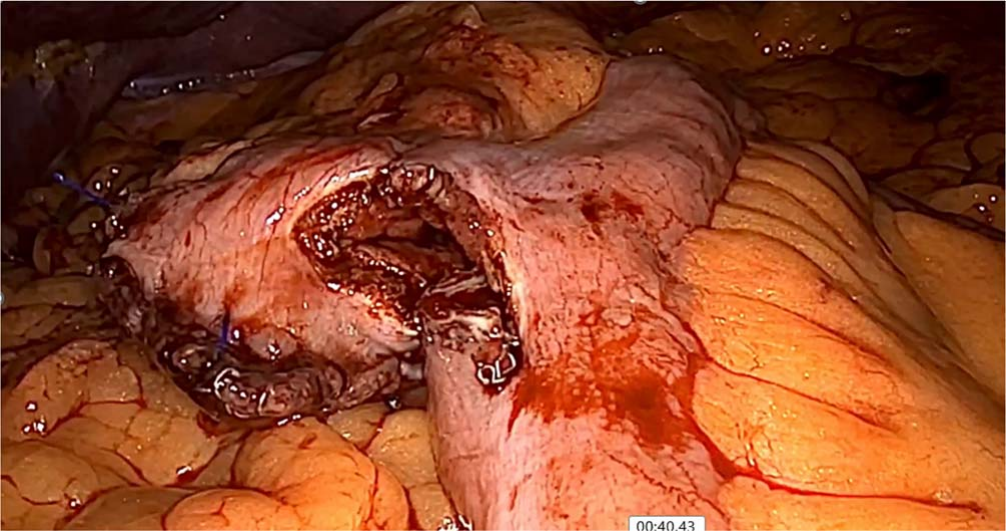
Isoperistaltic, antecolic, and side-to-side gastrojejunostomy is performed.

A 37-year-old man, with a body mass index of 49 kg/m^2^, who smoked for 20 years underwent OAGB without intraoperative complications (Fig. 1).

**Figure 2:**
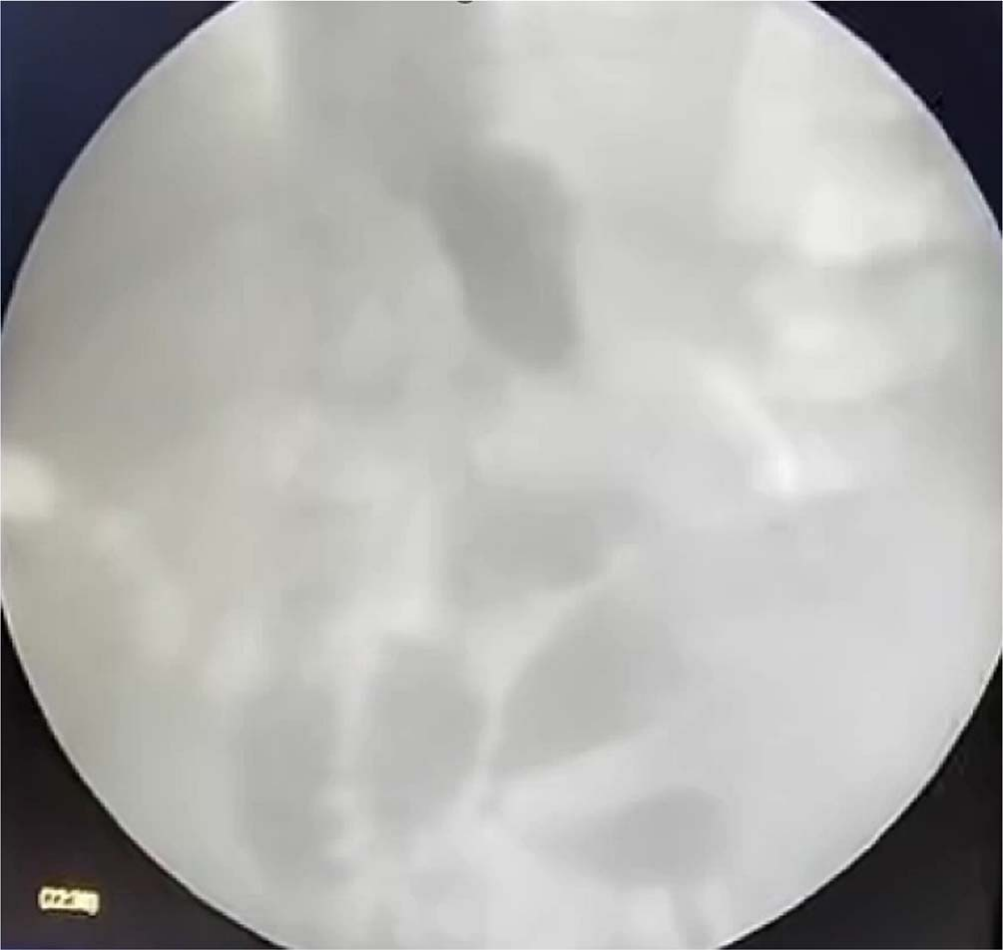
Fluoroscopy performed at 24 hours showing intraluminal contrast without leakage.

At 24 hours after surgery, fluoroscopy with water-soluble contrast media was performed (Fig. 2). The patient was discharged after 48 hours and treated with apixaban (orally every 12 hours) for thromboprophylaxis. Three days after surgery, he returned to the emergency room with tachycardia (130 bpm), nausea, vomiting, and severe diffuse abdominal pain on palpation. Laboratory tests revealed leukocytosis. Laparoscopic revision was performed.

**Figure 3:**
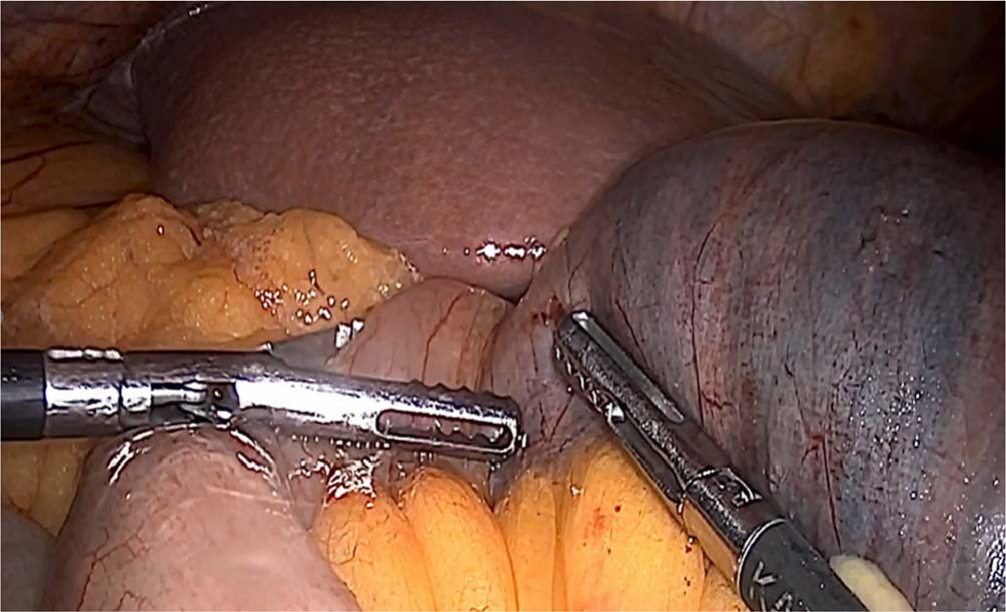
Biliopancreatic loop dilation.

**Figure 4:**
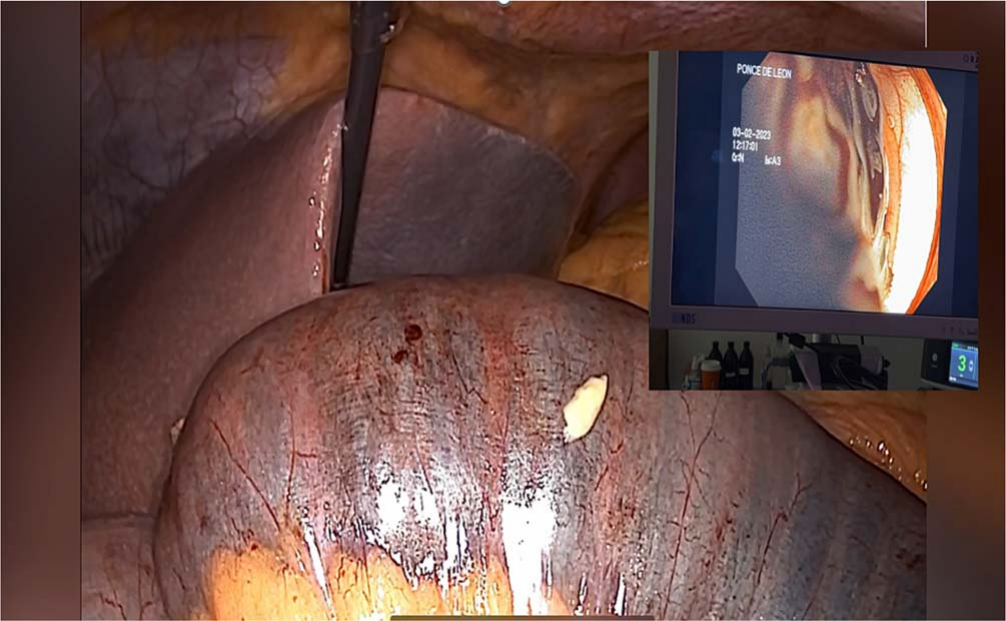
Endoscopy shows hemobezoar at the site of gastrojejunal anastomosis.

**Figure 5:**
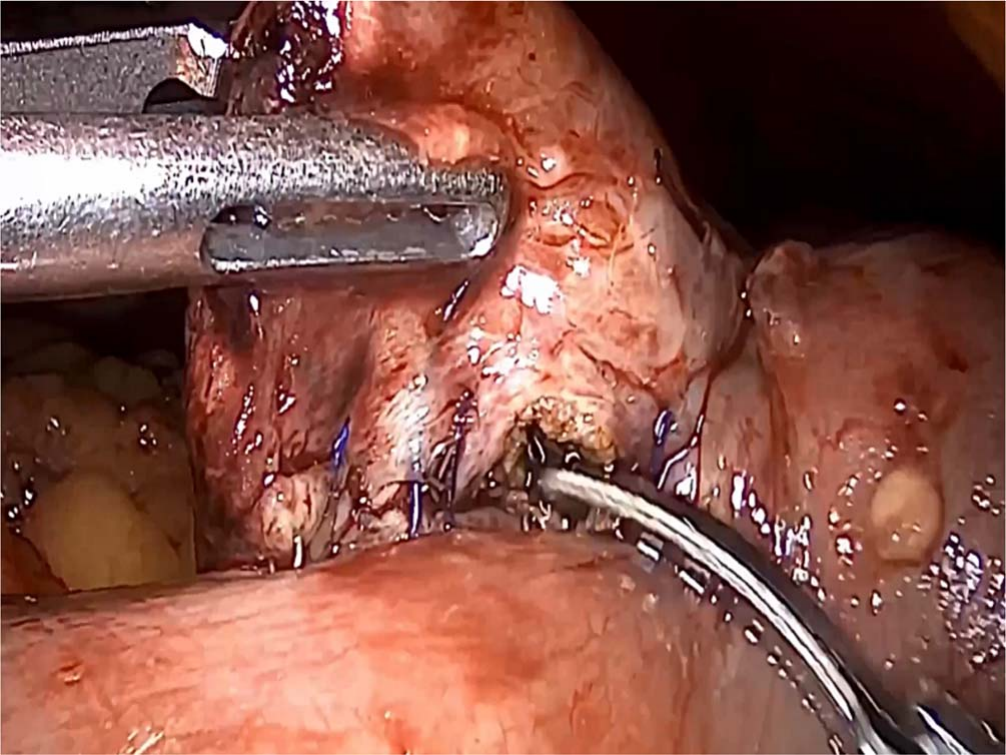
Removal of enterostomy suture.

**Figure 6:**
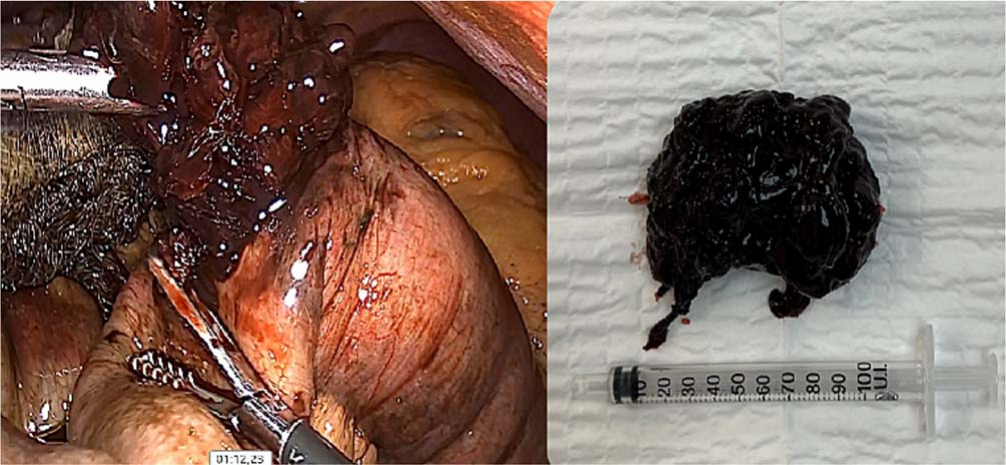
Hemobezoar removal.

**Figure 7:**
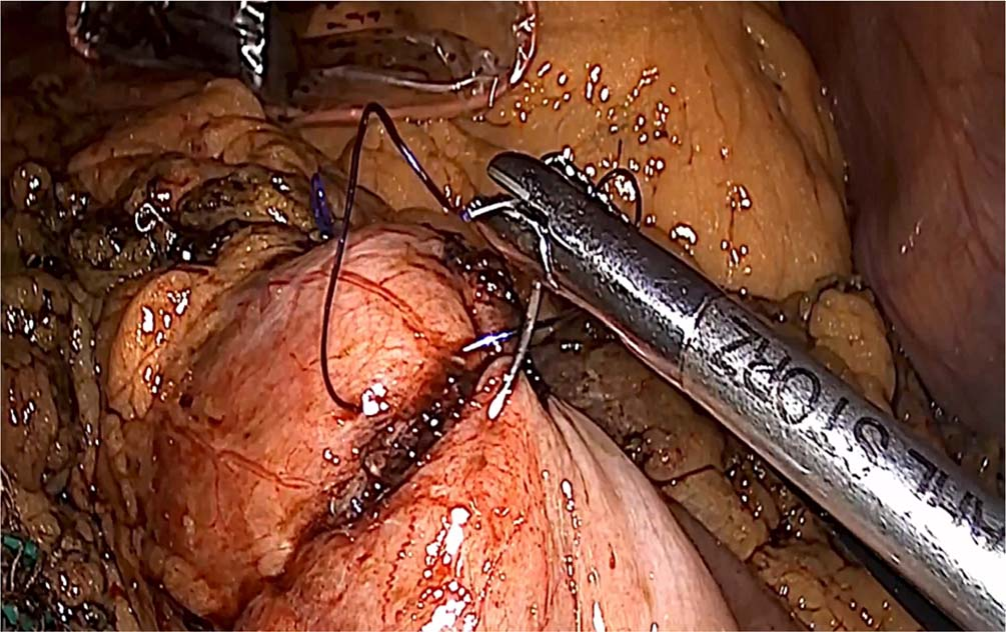
Closure of enterostomy in two planes.

A dilated biliopancreatic loop was identified during surgery (Fig. 3). Endoscopy showed an intestinal obstruction from a blood clot at the anastomotic site (Fig. 4). The previous suture was removed (Fig. 5). With delicate traction the hemobezoar was extracted (Fig. 6). A new enterostomy was performed, and the lesion was sutured in two planes (Fig. 7). A liquid diet was initiated 24 hours after surgery, and the patient was discharged 72 hours later without complications.

## Discussion

MGB/OAGB was introduced by Rutledge in 1997, and the term was established by the International Federation for the Surgery of Obesity and Metabolic Disorders. MGB/OAGB is the fourth most common surgery in Europe, providing several advantages and benefits to patients [1]. Nonetheless, MGB-OAGB can lead to complications, as observed in other types of bariatric surgery [2]. Early and late complications have been reported, with a mortality rate of 0.1%. The rate of early complications is 3.1%. Postoperative anastomotic leaks are the most life-threatening complications because of the presence of acidic and alkaline fluids.

Hussain *et al*. reported that three (0.3%) patients experienced bleeding in the anastomotic staple line of the gastric pouch. These cases required blood transfusion and laparoscopic control of bleeding with sutures. Endoscopic control of bleeding was attempted in one of these patients [2]. Musella *et al*. evaluated 2678 patients and identified one case of intestinal occlusion caused by a blood clot [1, 2].

Acute intestinal obstruction after MGB/OAGB is rarely caused by hemobezoar and also occurs after other surgical procedures, including Roux-en-Y gastric bypass. Hemobezoar can lead to severe complications if not detected early [3]. Symptoms include abdominal pain, vomiting, and abdominal distension [3, 4].

The early detection of potentially life-threatening complications, including anastomotic leaks, bowel perforation, and death, is essential [3]. Clot formation typically results from bleeding at the anastomotic staple line. Bleeding can be managed using minimally invasive enterotomy and surgical revision [2, 3]. Intraoperative endoscopy is useful for examining intraluminal mucosa and identifying the cause of the obstruction. Several factors can increase the risk of bleeding, including the learning curve, use of sealants, and longer operative time [1, 5, 6]. The Tri-Staple technology decreases the risk of bleeding. Enterotomy is an effective exploratory procedure that reduces the need for surgical revision [1, 5].

Anticoagulation is used as the first line for the prevention and treatment of venous thromboembolism in obese patients. The ISTH SSC published a guideline suggesting against the use of direct oral anticoagulants in patients with extreme obesity (body mass index >40 kg/m^2^ or weight > 120 kg) due to a lack of evidence. Bariatric surgery may affect the bioavailability of oral medications by decreasing the absorption surface area or reducing caloric intake. Apixaban absorption occurs primarily in the upper gastrointestinal tract (stomach and jejunum) [7].

Reduced oral caloric intake immediately after bariatric surgery may affect bioavailability in the acute dosing setting. However, specific data on the use of oral anticoagulants after bariatric surgery are scarce and limited to pharmacokinetic/pharmacodynamic studies with small numbers of patients or case reports [7].

In this context, it could be said that anticoagulation in our case may not be related to the bleeding and subsequent hemobezoar formation. Technical aspects of the procedure should be considered as the first option.

A hemobezoar was found at the anastomotic site in our patient during endoscopy. This condition led to intestinal obstruction. The suture was released, and the hemobezoar was removed laparoscopically. Postoperative recovery was unremarkable.

Diagnosing hemobezoar obstruction is challenging. It is a rare cause of complications, and few cases have been reported in the literature. In contrast to other reports, the initial diagnosis was not suspected of hemobezoar. Endoscopy and laparoscopy were used as diagnostic and treatment methods in the reported cases.

Not all intestinal obstructions after bariatric surgery are internal hernias, underscoring the need to perform differential diagnoses.
